# Can Cognitive and Behavioural Disorders Differentiate Frontal Variant-Frontotemporal Dementia From Alzheimer’s Disease at Early Stages?

**DOI:** 10.1155/2006/812760

**Published:** 2006-07-17

**Authors:** C. Jenner, G. Reali, M. Puopolo, M. C. Silveri

**Affiliations:** ^1^Memory ClinicCentre for the Medicine of the Ageing Catholic UniversityRomeItaly; ^2^ISSRomeItaly

**Keywords:** Frontal variant-frontotemporal dementia, Alzheimer's disease, memory deficit, executive deficit, behavioural disorders

## Abstract

Frontal variant-Frontotemporal dementia (fvFTD) and Alzheimer's disease (AD) patients matched for severity of dementia at the Clinical Dementia Rating (CDR) received neuropsychological testing in order to explore if the dysexecutive disorder might characterise fvFTD at early stage, when AD is dominated by the episodic memory defect. We also determined if the behavioural syndrome was more severe in fvFTD than AD, and if specific patterns of behavioural symptoms could differentiate the two types of dementia, using the Neuropsychiatry Inventory (NPI). AD patients performed worse than fvFTD not only in memory but also in executive tasks. Apathy and eating disorders proved to be more severe or frequent in fvFTD even if the two groups did not differ in the total NPI score. CDR score significantly correlated with the NPI score in fvFTD and with the MMSE in AD. Our data confirm that the memory disorders may differentiate the two types of dementia; however, the dysexecutive syndrome is as severe, and even more severe in AD. The severity of the behavioural syndrome is comparable in the two groups but the nature of the behavioural disorders may vary to some extent. We conclude that AD dementia at early stage is a behavioural-cognitive syndrome, while in fvFTD the behavioural disorders appear when the cognitive deficit is still relatively mild.

## 1. Introduction

One hundred years after its first mention and ten years after publication of the consensus clinical diagnostic criteria for frontotemporal dementia (FTD) [45], the clinical diagnosis of the frontal variant (fvFTD) still represents a challenge for the clinician. As laid out in the diagnostic criteria [30,45], and largely confirmed in the literature, deterioration in social behaviour and changes in personality constitute the core diagnostic features [1,14,45]. Although there is still inconsistent evidence regarding the diagnostic specificity of the behavioural syndrome in this pathology, it appears to be more frequent and more severe than in other forms of dementia (especially in AD) and to occur earlier [27]. Stereotypes, altered eating behaviour, dishinibition and changes in social conduct have been found to be more frequent or more severe in FTD [1,4,15,17,21].

The cognitive profile in fvFTD is not also well specified, whereas it is currently accepted that AD is defined primarily in terms of a distinct pattern of cognitive deficits. According to the currently accepted diagnostic criteria [30,45], fvfTD is characterized by the relative preservation of memory, visual and spatial abilities compared to a predominant impairment in executive functions. While some studies confirm the superiority of fvFTD patients on tests of episodic memory and/or visual and spatial ability [2,8,11,32,37], others did not

ISSN 0953-4180/06/$17.00 © 2006 – IOS Press and the authors. All rights reserved find significant differences between the two dementia groups [13,27,33,38]. There is also inconsistent evidence regarding prevalence and specificity of the executive disorders. Several studies failed to detect differences on traditional frontal executive tasks between fvFTD and AD [8,27,31,32], whereas others confirm a more severe impairment in fvFTD [10,13,22,34,37].

The aim of our study was to contrast the cognitive and behavioural profiles of patients with fvFTD and AD with mild-to-moderate dementia by using a multidimensional approach. First, we explored whether a dysexecutive disorder might characterise fvFTD at a stage of the disease evolution in which AD is dominated by the episodic memory deficit. Further, we aimed to verify whether the behavioural syndrome is more severe in fvFTD than AD, and whether specific patterns of behavioural symptoms could differentiate the two types of dementia.

## 2. Methods

### *2.1. Subjects*

Twenty-four patients with fvFTD and 22 patients with AD selected by standard criteria [25,30,45], as well as 22 healthy volunteers were included in the study (Table 1). Patients were referred for dementia assessment at the Neurology Unit of the Centre for the Medicine of the Ageing at the Catholic University of Rome, and received routine diagnostic assessment including physical examination, laboratory investigations, neuropsychological testing and anamnestic interviews with caregivers. All patients underwent structural neuroimaging, using either CT or MRI scan, and most fvFTD patients also had functional neuroimaging using HMPAO-SPECT. All subjects included within the fv-FTD group had evidence of anterior damage at least in one of the neuroimaging studies. Patients presenting symptoms of semantic dementia or primary progressive aphasia were excluded from the study as well as dementia patients with atypical onset (isolated or prevalent apraxia or agnosia) or with extrapyramidal signs.

We did not calculate the statistical power of sample sizes, since we were reasonably confident that for an exploratory study the number of patients studied would be adequate to show clinically significant differences. Moreover, there was neither "a priori" primary variable nor a fixed difference to test. In this context, negative results are less important than positive results. Considering the difficulties encountered when trying to evaluate dementia severity in FTD on a purely cognitive ground, severity was rated using the Clinical Dementia Rating Scale (CDR) [16] that takes into account not only memory disorders but also functional impairment. The functional status was also evaluated by the Activities of Everyday Living (ADL) and Instrumental Activities of Everyday Living (IADL). The MMSE [9] was used to assess the general cognitive impairment. All subjects gave informed consent for the inclusion in the study.

FvFTD and AD patients were matched by age, education and dementia severity, as expressed by the CDR (Mann-Whitney = 247.5; n.s.). All patients had a CDR score between 1 and 2. The ADL and IADL were also comparable. Controls were matched with patients by years of education. Controls and fvfTD patients did not differ by age; AD patients were older than controls (AD vs. controls: Mann-Whitney = 133.5; $p = 0.01$).

### *2.2. Tasks and procedures*

All participants were given a standard neuropsychological test-battery as well as tests assessing executive functions and memory. Due to the number of tests devised, testing took place in two or three sessions. The executive functions were investigated by using tasks traditionally supposed to explore the basic cognitive functions attributed to the frontal lobe: phonemic and semantic verbal fluency tasks (planning, initiating and set maintenance), short version of the Wisconsin Card Sorting Test and the Trail Making Test (part B) (TMT-B) (mental flexibility), Stroop-paradigm (selective attention and freedom from distractibility), Raven's Coloured Progressive Matrices (abstract reasoning). Moreover, two tasks originally taken from Luria's neuropsychological investigation were included: a tapping task, examining initiation and motor self-regulation, and a drawing task, evaluating the elaboration of complex syntax, rule-observation and distractibility. Memory functions were assessed by episodic memory tasks such as immediate and delayed free recall of 15 words (Rey Auditory Verbal Learning Test), Babcock story recall test, Rey-Osterrieth Complex Figure Recall, as well as by a Spatial Memory Test. Naming skills (semantic memory), ideomotor and oral praxis as well as visuo-spatial skills were also assessed.

A number of fvfTD and AD patients were not able to perform all higher-level executive tasks, particularly the WCST and the TMT-B (25% of fvFTD compared to 27.27% of AD-patients, and 12.5% of fvFTD patients compared to 77.27% of AD patients, respectively) due to inability to comprehend complex task instructions or mental inflexibility. We decided to assign to these subjects the worst score observed, on those particular tasks, in the group of dementia patients they belonged. The worst score was the most plausible value to be assigned to these patients, since the inability to perform the tasks was generated by the severity of the deficit in that particular cognitive domain. On the other hand, a bias might be introduced by excluding subjects unable to perform the task, that probably were, for the severity of the disorder, the more representative of that specific pathological condition.

The behavioural disorders were examined by means of the Neuropsychiatric Inventory (NPI) [6]. Although more specific behavioural scales have been proposed to explore the frontal lobe behavioural syndrome [19], the NPI was chosen in order to obtain comparable information both in FTD and in AD. In addition, since it is widely used, it tentatively allows comparing results obtained in different studies. It was administered through interview of primary caregivers, usually the patient's spouse or a child, by a clinician.

### 2.3. Statistical analysis

Comparisons between experimental groups and controls were carried out by the Mann-Whitney U test. To control for the probability of committing type I error in multiple comparisons, the Bonferroni correction was adopted setting p-value at $\leqslant 0.016$. Frequency distribution was analysed by the Fisher exact test. Correlations were performed by the Spearman rank order test. To identify which variables better discriminated the two dementia groups, a multivariate logistic regression with a stepwise forward procedure was carried out.

## 3. Results

### 3.1. Cognitive deficit: Executive and memory tasks

The performance of patients and controls is reported in Table 2. The scores obtained in some executive tasks, and in particular the TMT-B, Time-of-execution-component of the Stroop task and Drawing, were significantly worse in AD than in fvFTD (TMT-error: Mann-Whitney $= 149.5 -p < 0.0065$; TMT-time: Mann-Whitney $= 80.5 -p < 0.0000$; Stroop task: Mann-Whitney $= 84.5 -p < 0.0001$; Drawings: Mann-Whitney $= 396.5 -p < 0.0014$). AD patients performed worse than fvFTD also in episodic memory tasks: Immediate recall of words (Mann-Whitney $= 425.5 -p < 0.0000$); Delayed recall of words (Mann-Whitney $= 479.5 -p < 0.0000$); Babcock story recall test (Mann-Whitney $= 385.5 -p < 0.0053$). Also the Spatial memory was more impaired in AD (Mann-Whitney $= 400.5 -p < 0.0009$). No other difference reached statistical significance.

Controls performed significantly better than AD in all the tasks. FvFTD did not differ from controls in the Block span (forward), Time-of-execution- component of the Stroop task and in the Drawing. In all the other tasks fvFTD obtained significantly lower scores than controls.

A multivariate logistic regression with a stepwise forward procedure was carried out to identify which neuropsychological variables better discriminated the two dementia groups. The analysis selected two variables, a memory task (Delayed recall of words: RR $=$ 00; 95% CI $=$ 0.00–0.37) and an executive task (Stroop time of execution: RR $=$ 1.02; 95% CI $=$ 1.00–1.03).

### 3.2. Behavioural disorders: NPI

The total score obtained by fvFTD was higher compared to AD, but the difference did not reach significance. Only "apathy" was significantly more severe in fvFTD compared to AD (Mann-Whitney $= 408.5$; $-p < 0.0012$). No other difference reached significance. The distribution of the various pathological symptoms in the two dementia groups was also analysed after dichotomising the scores in normal and pathological (a behavioural domain was considered pathological when eliciting a total score higher than 0). The total distribution of normal and pathological scores was not significantly different in the two groups. Only "apathy" and "eating disturbances" were significantly more frequent in fvFTD than in AD (apathy: 23/24 vs. 12/22; Fisher exact test (two-tailed): $p < 0.004$); eating disturbances: 17/24 vs. 9/22; Fisher exact test (one-tailed): $p < 0.04$). No other distribution was significant. A logistic regression analysis performed on the dichotomised values ("0" and "$>$0") confirmed the significant value of the symptom "apathy" in discriminating the two dementia groups (RR $=$ 0.00; 95% CI $=$ 0.00–0.49).

### 3.3. Correlation between CDR and severity of cognitive and behavioural disorders

The CDR significantly correlated with the severity of the behavioural disorder (NPI) but not with the severity

Table 1
Demographic and main clinical data (mean and standard deviation)

| | fvFTD ($N = 24$) | AD ($N = 22$) | Controls ($N = 22$) |
|---|---|---|---|
| Age (yrs) | 73.37 (8.45) | 74.45 (9.52) | 68.95 (6.11) |
| Education (yrs) | 9.42 (4.80) | 7.91 (3.50) | 10.77 (4.69) |
| MMSE | 23.67 (3.02) | 19.59 (3.33) | 29.27 (0.83) |
| ADL | 5.12 (1.51) | 4.95 (1.17) | |
| IADL | 4.29 (2.83) | 4.04 (1.73) | |
| CDR | 1.46 (0.51) | 1.59 (0.73) | |

of the cognitive deficit (MMSE) in fvFTD (Spearman rank order test: CDR vs. NPI (total score) $= 0.4418$ $-p < 0.05$; CDR vs. MMSE $= -\ 0.2983$; $-p$ n.s.). An opposite pattern was observed in AD. In this group the CDR significantly correlated with the severity of the cognitive deficit (Spearman rank order test: CDR vs. MMSE $= -0.4397 - p < 0.05$) but not with the severity of behavioural disorder (Spearman rank order test: CDR vs. NPI (total score) $= 0.16$; ns).

## 4. Discussion

Our main results are the following: the two groups differed in severity on both episodic memory and executive functioning, with AD group scoring worse on both kind of tasks. The behavioural syndrome tended to be more severe in fvFTD, but statistical significance was only reached in the severity of "apathy" and in the distribution of "apathy" and "appetite disorders" (both more frequent in fvFTD). The CDR significantly correlated with the severity of cognitive disorder in AD and with the severity of behavioural disorder in fvFTD.

### *4.1. Cognitive deficit*

Our study corroborates the broad body of evidence indicating a more severe episodic memory impairment in Alzheimer's patients compared to fvFTD at mild to moderate stages of the disease [11,22,32]. The better performance of fvFTD in the Spatial memory task, also supports the superiority of these patients in visual and spatial abilities [2,8,11,32,37]. More surprisingly, AD performed at the same level or worse than fvFTD in executive tasks, confirming some previous reports [33, 41] but in contrast with others reporting a more severe executive impairment in fvFTD [10,13,22,34,37,41].

Several factors may account for these contrasting findings. In some studies FTD and AD groups are matched for severity of the cognitive disorders referring to their MMSE-scores. However, the MMSE may not be a satisfactory instrument to reliably rate severity of frontal dementia [23,42]. Especially in early and moderate stages patients may primarily present alterations in behaviour and personality being relatively preserved in cognition. Matching fvFTD patients with AD on the basis of the MMSE score might therefore imply selection of fvFTD patients who are by definition at more severe stages of the disease and then are presumably affected by more severe executive deficits. At the same time as outlined by Perry and Hodges [33] dysexecutive/attentional disorders seem to be the rule also in AD from the early stages and cannot assume reliable discriminative value. In addition, in spite of matching the general criteria for fv-FTD, patients might be potentially heterogeneous, due to a differential impairment of the various cortical – sub cortical circuits that constitute the neural substrates of cognition in the frontal lobe [26].

Our data basically confirm that the distinctive cognitive feature of fvFTD consists in the relative preservation of episodic memory more than in the severity of executive disorder. However, traditional "frontal" tasks may not be appropriate for detecting executive deficits specific of fvFTD. The information processing relying on the prefrontal cortex could be too abstract to become evident in conventional tasks mapping into perceptual input or motor output [39]. The so-called executive tasks are probably good tools to explore the functionality of the dorsolateral circuits, but less sensitive to capture cognitive deficits generated by a damage of the orbitofrontal circuits that would mostly express in terms of alteration of social cognition [5,26]. Less "conventional" frontal tasks such as "decision making" for example, demonstrated higher sensitivity to detect cognitive disorders following damage in the prefrontal cortex typical of fvFTD in early stages, when tasks assessing planning or working memory are still normal [35]. "Theory of mind" tasks (that explore the ability to understand and predict the behaviour of other people) [39], could also represent an efficient testing strategy to investigate specific aspects of social cognition in patients with frontal lobe damage [21,43].

Table 2
Neuropsychological performance of patients and controls (mean and standard deviation)

| | FTD ($N = 24$) | AD ($N = 22$) | Controls ($N = 22$) |
|---|---|---|---|
| *Memory* | | | |
| Digit span forward[a,*] | 5.08 (1.38) | 4.95 (0.95) | 5.54 (1.30) |
| Digit span backward[a,*] | 2.87 (1.29) | 2.50 (1.34) | 3.82 (0.85) |
| Block span forward[*] | 3.70 (1.76) | 3.68 (1.09) | 4.86 (1.17) |
| Block span backward[a,*] | 2.41 (1.50) | 2.59 (1.18) | 4.04 (1.17) |
| Immediate recall[a,b,*] | 21.37 (5.70) | 13.82 (6.19) | 46.77 (9.89) |
| Delayed recall[a,b,*] | 2.79 (1.89) | 0.18 (0.50) | 9.27 (3.53) |
| Babcock story recall[a,b,*] | 4.13 (3.46) | 1.58 (2.67) | 12.16 (3.10) |
| Rey-Osterrieth figure[a,*] recall(visual memory)[a,*] | 2.33 (2.29) | 0.45 (1.10) | 17.36 (6.70) |
| Spatial memory[a,b,*] | 11.21 (7.13) | 6.48 (6.62) | 24.59 (4.24) |
| *Semantic tasks* | | | |
| Naming nouns[a,*] | 24.46 (4.01) | 19.95 (6.50) | 27.86 (1.49) |
| *Praxis* | | | |
| Ideomotor[a,*] | 9.42 (0.65) | 9.04 (1.00) | 9.91 (0.29) |
| Oral[a,*] | 9.17 (0.92) | 9.00 (1.02) | 9.77 (0.43) |
| *Visuospatial tasks* | | | |
| Rey-Osterrieth figure copy[a,*] | 18.17 (12.47) | 13.20 (12.43) | 33.41 (2.84) |
| *Executive tasks* | | | |
| TMT-B time[a,b,*] | 454.79 (270.53) | 789.50 (214.22) | 180.09 (68.81) |
| TMT-B-errors[a,b,*] | 11.92 (7.49) | 17.45 (7.03) | 1.10 (1.31) |
| WCST-categories[a,*] | 1.33 (1.34) | 0.78 (0.87) | 4.41 (1.62) |
| WCST-errors[a,*] | 24.50 (12.42) | 26.95 (11.87) | 10.59 (3.63) |
| Semantic fluency[a,*] | 30.50 (12.14) | 22.83 (11.35) | 67.45 (18.80) |
| Phonological fluency[a,*] | 17.25 (9.28) | 12.23 (8.66) | 40.45 (13.39) |
| Raven's CPM[a,*] | 16.83 (5.54) | 13.18 (8.19) | 28.91 (4.32) |
| Attentional Matrices[a,*] | 44.75 (10.95) | 40.18 (14.70) | 53.72 (5.97) |
| Stroop-time[b,*] | 102.79 (147.93) | 187.27 (114.14) | 74.27 (50.70) |
| Stroop-errors[a,*] | 14.50 (11.89) | 13.14 (6.03) | 1.54 (1.94) |
| Drawing task[b,*] | 3.34 (2.65) | 0.90 (1.85) | 5.00 (1.15) |
| Tapping task[a,*] | 5.12 (4.16) | 3.86 (3.76) | 9.82 (0.50) |

Significant difference (Mann-Whitney $p \leqslant 0.016$):
[*]between controls and AD.
[a]Between controls and fvFTD.
[b]Between fvFTD and AD.

### 4.2. Behavioural disorders

FvFTD patients showed more severe and/or more frequent symptoms of apathetic behaviour and eating disorders than AD patients. The prevalence of apathy finds substantial support in the literature [1,3,19–21, 24]. Apathy has been traced back to lesion of one of the frontal subcortical circuits, the anterior cingulate circuit [5,44] that also could account for eating disorders [1,4,24,32]. Thus, our results are consistent with previous reports regarding two of the more commonly reported behavioural disorders in FTD. However, we should acknowledge that without autopsy-based confirmation [37], our results, like those of other purely clinical studies, could be tautological to some extent, since selection criteria may include these parameters. At the same time we were unable to confirm group differences in other behavioural domains that have been reported in other studies. In general, heterogeneity of the reports is large and to date, no unitary account of the behavioural syndrome of FTD has been provided. Heterogeneity may be partially accounted for by methodological factors. Different behavioural scales have been adopted in the various studies so that results may not be fully comparable. The NPI that we used is probably less sensitive than other scales in detecting behavioural abnormalities typical of fvFTD, which could have led us to underestimate the presence and severity of some symptoms (for example stereotypic behaviour). However, the choice of the NPI was justified by our decision to use a commonly used scale that could be also applied to an AD population. Indeed, this scale proved to be able to capture at least some of the behavioural differences between these two populations of dementia patients.

Differences between groups in the distribution of the neural damage in the frontal lobes [21] might account

Table 3
NPI scores in the dementia groups (mean and standard deviation)

| | fvFTD ($N = 24$) | AD ($N = 22$) |
|---|---|---|
| NPI | | |
| Delusions | 0.87 (1.87) | 2.00 (3.96) |
| Hallucination | 1.00 (2.43) | 0.54 (1.94) |
| Agitation | 3.00 (3.37) | 2.04 (3.11) |
| Depression | 4.04 (2.40) | 3.41 (3.89) |
| Anxiety | 2.21 (3.37) | 1.14 (1.86) |
| Euphoria | 0.21 (0.83) | 0.23 (0.87) |
| Apathy* | 6.46 (4.16) | 2.82 (3.54) |
| Disinhibition | 1.29 (3.03) | 0.77 (2.65) |
| Irritability | 2.17 (3.38) | 2.27 (2.69) |
| Aberrant motor behaviour | 1.08 (2.30) | 1.27 (3.28) |
| Sleeping disorders | 2.75 (4.02) | 2.18 (3.02) |
| Appetite disturbance | 3.50 (3.93) | 2.32 (3.47) |
| TOTAL | 27.62 (21.52) | 20.50 (18.98) |

*Significant difference (Mann-Whitney) at $p < 0.0012$.

for other behavioural differences. Additionally, laterality appears to be a relevant factor of variability, yet it is only occasionally considered.

### *4.3. Conclusion*

Our data suggest that in early stages AD is basically a cognitive-behavioural syndrome, dominated by the episodic memory disorders, whereas the behavioural syndrome may be evident in fvFTD when the cognitive deficits are still relatively mild and do not fit any neuropsychological model of dementia. Apathy and eating disorders seem to be distinctive features of the behavioural syndrome in our group of fvFTD, at least among the behavioural symptoms that the NPI is able to capture.

The frontal-subcortical circuits represent the neural substrates of functions that cannot be easily distinguished in terms of cognition and behaviour, and this probably generates some inconsistency. Possibly, damage to the dorsolateral circuit may be quantified by the severity of planning or attentional defects. However, translating the reduced functionality of the orbitofrontal or cingulate circuits to abnormalities on testing is much more difficult and requires application of specific testing procedures sensitive to changes in social behaviour. In other words, distinction between cognition and behaviour might be to some extent artefactual. Measures of cognitive dysfunction, especially reduced ability in planning tasks, predicted the severity of psychiatric disorders measured by the NPI in fv-FTD but not in AD [40]. Newly proposed testing techniques might be more appropriate to explore components of the "frontal syndrome" that manifest as disorder of social behaviour and personality changes [12,29,43]. For example, a significant correlation has been found between "Theory of Mind" (ToM) tasks such as the "false belief" and the "faux pas" test and the NPI, suggesting that cognitive tasks and neurobehavioral scales may measure a common process [12]. Further work is required to confirm this hypothesis. Neurimaging studies could also contribute to disentangling the role that the various cortical-subcortical circuits play in the complex syndrome generated by the frontal damage [7,18,46].
